# Acceptability and Feasibility of Portable Eye-Tracking Technology within a Children’s Dynamic Sport Context: An Exploratory Study with Boys Who Play Grassroots Football

**DOI:** 10.3390/sports12080204

**Published:** 2024-07-26

**Authors:** Katie Fitton Davies, Theresa Heering, Matt Watts, Michael J. Duncan

**Affiliations:** 1 Research Institute for Sport and Exercise Sciences, Liverpool John Moores University, Liverpool L3 3AF, UK; k.fittondavies@ljmu.ac.uk; 2 Centre for Physical Activity, Sport and Exercise Sciences, Coventry University, Coventry CV1 5FB, UK; theresa.heering@med.uni-duesseldorf.de (T.H.);; 3 Heinrich Heine University, 40225 Dusseldorf, Germany

**Keywords:** motor learning, vision, sports, perception, football, motor skill

## Abstract

Teaching practices are moving from decontextualised to more representative curricula. Although this is argued to be a positive step, low motor competence is a continual issue in primary-aged school children. One methodological approach to investigate ways to improve motor competence, eye tracking, is moving to more representative tasks. So far, eye-tracking research using static activities has demonstrated a positive association between motor competence and earlier fixation and longer duration. However, this research has been constrained to laboratory settings and tasks, or discrete activities (e.g., throw and catch). This study seeks to understand how to conduct more representative eye-tracking research in primary school-aged children. To this end, thirteen 10–11-year-old children were fitted with an eye-tracker during a typical football coaching session. Children were asked acceptability-based questions, and eye-gaze data were captured to illustrate what children attended to under a representative dynamic football-based activity. Based on the voices of children and captured eye-gaze data, six practical implications for research in this population are proposed: (1) conduct eye-tracking research indoors (where possible); (2) ensure long hair or fringes are secured so as not to obscure line of sight; (3) run the same activity to increase comparability across children wearing the eye-tracker; (4) use a properly fitted backpack (if a backpack is to be used); (5) assure children about the capability and hardiness of the eye-tracker, as they do not need to change the way they move; (6) explain there may be some discomfort with the nose clip, head strap, and battery weight and ensure that children wish to continue.

## 1. Introduction

It is well established that a higher level of motor competence (i.e., goal-directed human movement [1]) is associated with many positive outcomes in children as they mature [2,3]. These positive outcomes include improved fitness [2,3], physical activity [1,2,4], cardiorespiratory fitness and healthier weight status [1,2,4] and executive function [5]. However, although better motor competence during childhood is a pathway to a more positive developmental trajectory (in comparison to lower motor competence), studies have consistently demonstrated that levels of motor competence in children are below the expected standard [6,7,8,9]. One of the main aims of the National Curriculum for physical education (PE) in England is to “…develop fundamental movement skills, become increasingly competent and confident and access a broad range of opportunities to extend their agility, balance, and coordination, individually and with others” ([10], p. 199). However, it seems that children may not be receiving adequate support to help develop motor competence in PE. Yet, this inference of a lack of sufficient support is not, seemingly, through a lack of trying on behalf of the teachers.

The use of differing pedagogical approaches has demonstrated variable effects on an aspect of motor competence, namely fundamental movement skills (FMS), such as running, jumping, kicking and catching [11]. For example, studies have investigated the effect on FMS through teacher-led traditional games [12], teaching underpinned by achievement goal theory [13], a multi-skills club [14], specialist PE-led [15] and nonlinear pedagogy [16]. Nevertheless, there may be another individual-based indicator that teaching, and instruction, can tap into, which has been largely overlooked but has recently started to gain momentum: eye gaze.

From an evolutionary perspective, eye-gaze behaviour provides the necessary information to guide our ancestors through their environments, enabling them to act as needed (e.g., viewing an animal to throw a spear at) [17]. As such, eye-gaze behaviour is inherently intertwined with an individuals’ ability to inform and successfully execute a goal-directed motor task. Research has demonstrated that children with higher levels of motor skill also have superior visuomotor control [18]. Taking this research further, Miles et al. [19] found that children who undertook quiet eye training improved their throwing and catching performance compared to children who did not undertake the quiet eye training. This performance difference and difference in visual gaze aligns with research in the intermediate [20] and elite athlete literature [21,22].

In a bid to examine eye-gaze behaviour in more representative environments (in comparison to lab-based studies), Aksum et al. [23] investigated the visual fixations of 17–23-year-old elite footballers. By fixing mobile technology (i.e., Tobii Pro Glasses) on the players during a match, Aksum et al. found that elite footballers used longer fixations when there were more areas of interest in the fixation circle (e.g., ball, opponent, teammate). Furthermore, the research designs researchers use to explore visual fixations may alter the search strategies that individuals use. Therefore, using representative designs which replicate the match context may provide more accurate information around visual search strategies in comparison to decontextualised study designs, such as those employed in lab- or drill-based contexts. So far, eye-tracking studies have been successfully undertaken in older populations (primarily adults) in basketball [24], ice hockey [25] and futsal [26]. Understanding how eye gaze develops in younger children may be useful for informing teaching practice, and consequently, improving children’s motor skills. However, to date, research has not examined whether children can effectively wear eye-tracking technology and whether it is feasible to obtain eye-tracking data during tasks representative of sports performance. The use of portable technology has its obvious benefits for research (e.g., unconstrained from laboratory, use in dynamic movement rather than remaining static); however, from a practical application reality, it may pose potential problems for younger age groups. For example, a potential issue may be the weight of the technology or interference on movement production. There are yet no data on how acceptable the use of eye tracking might be in children or how feasible it is to conduct this type of research in this population. This is a key first step in establishing process for use of eye-tracking technology in sports with paediatric populations.

This is important as studies examining how eye-gaze behaviour relates to other aspects of movement or sport performance in children, e.g., motor competence, are lacking. The most recent systematic review [27] on the topic only identified three papers conducted with children; two previously mentioned in this current paper [18,19] and one that investigated quiet eye training in children with developmental coordination disorder [28]. Since that review, few studies [29,30] have been conducted using eye tracking with children, and no study to date has examined the acceptability of using the technology with its participants. As a result of this aforementioned literature, it is clear that children can use eye-tracking technology in relatively static learning and performance environments. However, in the context of dynamic situations, such as those in any movement task or sport, whether eye tracking is feasible in children remains an unresolved issue. Exploring the utility of eye-tracking technology with children in dynamic movement situations is a key first step to establishing to what extent this technology can aid research in this area and identifying best practices for the use of eye-tracking technology in children during sport and physical activity. Importantly, no study to date has included the children’s voice in understanding how acceptable eye tracking is from the perspective of those using this technology.

We are inhabiting an era where motor development research is moving away from the traditional approach to learning (e.g., Direct Instruction Model [31]), which primarily focuses on repetition of skill through drills. Instead, other, more learner-centred pedagogical approaches are becoming popular, such as Teaching Games for Understanding [32] and the constraints-based approach [33]. To that end, it is important to understand what children attend to within these more dynamic and representative learning environments. As individuals with better motor competence have significantly different visual fixation strategies in comparison to individuals with worse motor competence [18,19], perhaps there are lessons to be learned on how to best support PE instruction. However, due to the lack of research in younger populations, guidance on how best to conduct this research within this younger age group has not been established. Although there are guides in existence for researchers and eye-tracking research in general [34], particular guidance for research with children is lacking. Given that there remains contradictory results (see [27,28,29,30]) relating to the importance of eye-gaze behaviour in executing motor tasks in children, and that the acceptability of eye-tracking in children has not been established prior to researchers conducting studies using eye-tracking technology with children, it may be that the design of such studies has not fully considered how children experience the process of using eye tracking. This could be one reason why there are contradictory results in the literature examining eye tracking in children to date. Therefore, the main aim of this study was to explore the acceptability and feasibility of investigating eye-gaze behaviours of children in a representative, dynamic sport environment. This aim was pursued with two main questions: (1) Does the mobile technology work with children in a dynamic sport environment? (2) Is this form of research acceptable to children? These questions were answered through fitting the mobile technology and ascertaining the eye-gaze percentages and asking children a series of questions after wearing the mobile technology, respectively. Movement assessments were also conducted to more fully describe the children involved in the study. In addition, recorded eye gaze was analysed to provide some preliminary description as to what children in this context were attending to.

## 2. Materials and Methods

### 2.1. Study Design

This study was exploratory in design and sought to understand the acceptability of using eye-tracking technology with children in a dynamic sport setting. As such, the study used a participatory approach where children worked with researchers to understand, via qualitative data collection, the acceptability of the eye tracking in the context of a dynamic sport task. Analysis of the eye-tracking data obtained was of secondary interest and comprised a descriptive analysis to understand if the mobile eye-tracking technology produced useable data with children in this context.

### 2.2. Participants

Thirteen boys aged 10–11 years (mean ± SD, height = 145.34 ± 6.33, sitting height = 71.97 ± 3.61, mass = 38.02 ± 7.12) attended Coventry University’s sports facilities over two days. The boys were regularly engaged in grassroots football (i.e., were not part of a professional academy but participated in organised competitive football) and had, on average, 4.06 ± 0.10 years of football playing experience (range 2–5 years). Prior to participation, the project was given institutional ethics approval (protocol no. 131207), parents provided written informed consent, and children provided assent to take part. Inclusion criteria were being a child aged 10–11 years, who was registered with a grassroots football club and regularly engaged (having played for at least one year) in grassroots football.

### 2.3. Apparatus

#### Eye-Tracking

Each child wore the TobiiGlasses2 corneal reflection eye movement system (Tobii Technology AB; Danderyd, Sweden) to record their visual search behaviours while engaging in football-related practices. The battery pack was secured in a backpack to avoid dislodging the device while moving. Responses to acceptability questions were recorded via the microphone in the system. Before each child was able to participate with the glasses, the calibration procedure had to be administered. To this end, children were asked to fixate on the central spot of a playing card-sized target of concentric circles until the software registered the eye gaze. Each child wore the glasses for around 10 minutes. The data collated from the TobiiGlasses2 comprised the number of fixations and saccades, and the gaze duration for each which occurred for each participant during the time period that the TobiiGlasses were worn. From this, the object or objects that the participant was looking at during each fixation or saccade could be determined. Gaze fixations were defined as the maintaining of gaze on a single location/object (e.g., the ball), whereas shared gaze fixations occurred where gaze was maintained on more than one location/object at the same time (e.g., the ball and the ground). Data were stored on SD card within the TobiiGlasses battery pack and were subsequently downloaded for the analysis of gaze behaviour.

### 2.4. Measures

#### 2.4.1. Motor Coordination

Football-specific motor coordination was assessed using the Ghent University (UGent) soccer specific dribbling test [35]. In this assessment, participants run as fast as possible, without a ball, changing direction around eight cones in a pre-determined sequence (four to the left, four to the right at different angles). Distance between cones ranged from 1 to 2.20 m, and time taken from start to finish was measured with a stopwatch (0.01 s). Each participant completed the task twice, first without a ball, and second with a ball. Any participant who lost control of the ball (2 m away from trajectory) undertook a subsequent trial. Prior work has employed this assessment with the same age range of children in the present study [36].

#### 2.4.2. Fundamental Movement Skills (FMS)

Six movements from the Test of Gross Motor Development-3rd Edition (TGMD-3 [37]) were completed, split into locomotor (run, jump, hop) and object control (overarm throw, underarm throw, catch) and considered measures of FMS. Of note, the term FMS is used to refer to fundamental movement skills in the present study, as opposed to the Functional Movement Screen, which has also previously been abbreviated to FMS in other work. Children watched one demonstration per skill provided by the trained administrator and then proceeded to have one practice trial before completing two video-recorded trials, which were assessed. Children’s movements were scored on specific criteria (0 = not present, 1 = present). All skills were video-recorded and coded by a trained coder. The TGMD-3 is a reliable test of motor performance in children aged from 4 to 10 with inter- and intra-rater reliability above an Intraclass Correlation Coefficient (ICC) of 0.96 [38], which is considered excellent [39]. For the six movements, scores ranged from 0 (no criteria were present across two trials) to 46 (all criteria were present across two trials).

#### 2.4.3. Acceptability of the Eye-Tracking Device

To understand the acceptability of the eye-tracking device, in-depth techniques were used during questioning. Acceptability questions were underpinned by work from Alexandre et al. [40] who conducted a literature review on acceptance and acceptability of tools by users, resulting in a framework. Under this framework, questions for acceptability should include the following constructs: *Utility* (e.g., perceived usefulness, performance), *ease of use* (e.g., perceived ease of use, difficulty to perform for user), *contextual and social differences* (e.g., age, gender, active experience, concentration), *aesthetics* (e.g., image, visibility) and *overall judgement* (e.g., user satisfaction, intention). Utility was not judged to be relevant for this sample as wearing the mobile technology had no direct purpose for the children while completing the football-related tasks. Aesthetics were also omitted as we were not interested in long-term uptake. Therefore, at the end of 10 min of wear time, each child was asked a series of questions relating to ease of use (e.g., *Were there any issues while wearing the eye tracker? If so, what were they?*), contextual and social differences (e.g., *could you move how you wanted to while wearing the eye-tracker?*) and overall judgement (e.g., *How did you feel about the pack on your back? How did you feel about wearing the glasses? Would you be willing to wear it for longer during similar activities in future?*). 

#### 2.4.4. Procedure

Over two days, the ‘Coventry Young Footballers’ event was held in a sports facility which had predominantly outside astroturf playing spaces. During the morning session of both events, the children were assessed on their motor coordination and FMS. In the first event, during the afternoon, groups of 6–12 children took part in, essentially, a coaching session, which had the children complete a warm-up and a series of practices, and to finish, they took part in a small-sided game (i.e., conducive to a “typical” training session for them). The first child was fitted with the TobiiGlasses2 and completed the calibration procedure, which took less than five minutes to complete. The first child then wore the glasses for 10 minutes of playing time. Once 10 minutes was finished, the child was called over to the researcher and asked the acceptability questions while still wearing the glasses. Once the child had finished answering the questions, which took around two minutes to complete, the second child was called over. The visual data collection process was repeated throughout the coaching session until all children had participated.

### 2.5. Data Analysis

Note that the aim of the study was to determine acceptability of use of the eye-tracking device with children and, as such, our analysis focused on descriptive data of what children viewed and the data from acceptability interviews, rather than any inferential statistical tests; recognizing such an analysis would have been underpowered and was not the aim of the present study. Statistical tests were completed using SPSS, version 26 [IBM SPSS Statistics Inc., Chicago, IL, USA]. Descriptive statistics, including mean and standard deviation, were calculated for each variable and can be seen in Table 1. Acceptability interviews were transcribed verbatim and reported descriptively according to the sub-categories present by Alexandre et al. [40]. Details on gaze event type (e.g., fixation, saccades and unclassified) and gaze event duration were exported from TobiiGlasses2 Software (v.1.114) into Microsoft Excel. The gaze filter “Tobii I-VT (Fixation)” was applied to identify the children’s gaze focus for each fixation. The fixation focus was documented manually for the first five minutes of wear-time spend in “typical” training (e.g., excluding eye-tracker set-up time). The coding referred to the background (fence, trees), goal, opponent/goalkeeper, coach/researcher, ground, ball, sky, boundary (line, cones), self (own hand, leg) or unidentifiable.

## 3. Results

This section first presents the descriptive data of the participants’ characteristics (Table 1), then the acceptability data, followed by descriptive data of what children attended to during eye tracker wear time. Please note, a posteriori examination of eye-tracking data from the two time points where data were collected demonstrated no discernible differences in the pattern of gaze behaviours undertaken by participants. As such, data from the two timepoints of data collection are presented together.

### 3.1. Acceptability

The acceptability data are presented in accordance with the themes outlined by Alexandre et al. [40]: ease of use (perceived ease of use, user friendliness/usability, difficulty to perform for user, physical effort), contextual and social differences (active experience, concentration) and overall judgement (user satisfaction, intention).

#### 3.1.1. Ease of Use

Most children reported no issue with the eye tracker or the battery pack (perceived ease of use), which was housed in a backpack, rather than clipped to the children’s trousers/shorts. However, one child commented on the heaviness of the battery pack (“yeah it was a bit heavy”), while another commented on the bag being too loose. A couple of children commented on the frames obscuring their vision (“hard to see…like the frames’ in the way [did it stop you from seeing what you wanted to see?] no not really [you were just aware of it?] yeah”, “not really but it just like when I try and like look that’s like blocking it [when you try to look down?] yeah”). A couple of children expressed that they felt like they could not header the ball, despite being told at the start that they could (“I thought if I headered it I would break them”).

No discomfort was reported (user friendliness); however, one child felt “weird” about being able to see the lower frame while looking down, and another commented that the nose clip was pushing down their nose a little. There was a more mixed response when they were asked if the glasses or backpack made it difficult for them to move how they wanted to (difficulty to perform for user). While some children reported no issues, others remarked on the heaviness of the backpack and how it altered their movements (“it’s more heavier…had to change how I moved” “probably a bit slower so it doesn’t fall off or anything…the glasses are fine” “think maybe a bit slower because it means a bit more weight”). The backpack also caused the children to alter the way they held themselves (yeah…like when I was like moving and that like it felt different” “sometimes cos when I go like that the backpack moves” “yes had to make sure the pack was straight and had to alter body to do that”). When the children were asked directly if the backpack was too heavy for them (physical effort), 11 children reported that it was fine, while 2 reported that it was heavy or a little heavy. All but one child reported that the glasses themselves did not obstruct their vision when directly asked (difficulty to perform for user) (“…I was like this but if the ball’s there I can’t really see the ball that good [because of the bottom of the glasses] yeah”).

#### 3.1.2. Contextual and Social Differences

When the children were asked if they could move how they wanted to while wearing the eye tracker (active experience), all but two said yes. One child remarked on the looseness of the backpack, and one reported that they were mindful of being more careful “just in case”. Ten children reported no when they were asked if the glasses or backpack distracted them in any way (concentration). One child commented on the frames as a distraction (“when you were doing the little running backwards thing when you’re about to turn around it’s kind of hard to see [because of the frames] yeah”). Another child commented on the method of glasses attachment (“this bit on the back [back of the head] yeah [was it too tight] no it was just hanging [oh the dangly bit] yeah”). The third child commented on the backpack straps (“yeah cos when it kept sliding I had to put it back up”).

#### 3.1.3. Overall Judgement

Most questions were centred around user satisfaction as a sub-theme of overall judgement. When the children were asked how they felt about the pack on their back, most provided positive responses (“I honestly just forgot about it”), while one commented on the weight, and another commented on the sliding straps. The children were asked a similar question about the glasses, and most reported that the glasses were fine to wear. A couple of children expressed that it was “not too bad but not too good it’s like middle” and “it’s not that comfortable but it’s fine”, while another child felt a certain perplexity towards the glasses (“dunno just felt weird [bad weird or ok weird] ok weird”). When the children were asked if they would be willing to wear eye tracker for longer during the same activity or different activity in future (intention), 10 children said yes. Two children remarked that 10 min was enough, while another described that they would wear it for a different activity but only for another five minutes.

### 3.2. Gaze Fixations

During the data collection held during the first event, the lowest eye-gaze percentage was 48% and the highest was 90%, with a group average of 74.08%, and 6 of the 13 children obtaining an eye-gaze capture of 80% or over. Here, 80% was set as a threshold for usable data as a contingency for the natural variation of tasks for individuals within a soccer coaching scenario. When data were collected at the second event, the lowest eye-gaze percentage was 1% and the highest was 67%, with a group average of 27.67%; no child obtained an eye-gaze percentage over 80. This section also describes what the children were attending to when wearing the eye tracker during the first data collection period (Table 2). Coding five minutes of “typical” training took, on average, two hours with an average of 482 fixations (range: 441–563 fixations) per recording. The fixation focus was challenging to examine during fast movements (e.g., ball being passed). In those cases, for example, the fixation focus was “behind” or “beside” the ball but “followed” the ball through several fixations in a row.

Keeping in mind that of the 10 min wear time, the first 5 min was coded and only five children were coded of the 13 included in this study (a sub-sample of children was coded to provide information on time taken to code the data; to provide information for future research), Table 2 starts to present a profile-like display for the individual children. For example, although the number of fixations on *opponents* was high across the five participants, Participant 1 fixated highly on the *goal* (*n* = 136), while the other four participants did not fixate on this to such an extent (*n* = 14–50). Participant 4 fixated to a greater degree on the ball (*n* = 139), while, although the other four participants also fixated on the ball, this was done to a lesser extent (*n* = 41–72). These data demonstrate that although all children were involved in similar coaching sessions with similar intended outcomes, their attention was drawn differentially across the environment.

### 3.3. Shared Fixations

Table 2 illustrates that most of the children fixated on environmental objects like the *ground* and *background*, which were arguably less relevant to the PA task they were involved with. When looking at the data, there were several shared fixations (i.e., more than one type of object involved in the fixation).

Participant 2 had 80 occurrences of shared fixations (18.14% of total fixations). Overall, 68 of those 80 shared fixations were with the ball (85%). The primary focus of those 68 fixations with the ball was with the ground (73.53%). This relationship between the ball and ground made up 28.09% of total ground fixations for participant 2.

Participant 3 had 51 occurrences of shared fixations (11.23% of total fixations). Overall, 31 of those 51 shared fixations were with the ball (64.71%). The primary focus of those 51 fixations with the ball was also with the ground (72.73%). This relationship between the ball and the ground made up 18.18% of total ground fixations for participant 3.

Participant 4 had a more varied shared fixation profile. Participant 4 had 86 occurrences of shared fixations (18.61% of total fixations). Overall, 63 of those 86 shared fixations were with the football (73.25%). Of those 63 shared fixations with the ball, 41.27% were with the ground, 23.81% were with the background, and 22.22% were with the goal.

Participant 5 had 67 occurrences of shared fixations (13.73% of total fixations). Overall, 43 of those shared fixations were with the ball (64.18%). The primary focus of those 43 fixations with the ball was with the ground (62.79%). This relationship between the ball and the ground made up 18.24% of total ground fixations for participant 4. In contrast to the other four participants, participant 1 only had 4 shared fixations (0.71% of total fixations), with three of those shared fixations between the ball and background (75%).

## 4. Discussion

This study explored, for the first time, the acceptability and feasibility of investigating the eye-gaze behaviours of children during a dynamic sport task through two main questions: (1) Does the mobile technology work with children in a dynamic sport environment? (2) Is this form of research acceptable to children? More specifically, this study investigated whether the eye-tracking apparatus (TobiiGlasses2) would be acceptable to use in football-based scenarios with 10–11-year-old boys. As a result of this study, guidance will be provided on how to investigate eye-gaze behaviour in more dynamic environments with a younger population than has been previously investigated in the literature to date. The study also aimed to demonstrate details on the captured eye-gaze behaviour of these football players while wearing the technology, offering novel insights into this particular population of study.

### 4.1. Feasibility of Using Portable Eye-Tracking Devices in Children

To help answer the first question (does the mobile technology work with children in a dynamic sport environment?), we investigated the percentage of eye gaze captured at two time points. More specifically, this was investigated to ascertain the extent of data capture and data loss when wearing TobiiGlasses2 within dynamic football-based scenarios. The percentage of eye gaze was much higher in the first data collection time point in comparison to the second. This lower eye gaze capture at the second time point could be because although both data collection periods occurred outside, the weather was different. In May, the weather was overcast, which meant the sun rarely shone. In contrast, in August, there was no cloud cover, and the sun shone almost constantly. Sunlight can interfere with the infrared light that travels from the frames of the glasses to the eye pupil, meaning the technology fails to capture the eye-gaze behaviours under these conditions. Therefore, although the first data collection point demonstrated that most children provided enough captured eye gaze to be usable, **practical implication 1** would be to conduct this type of research indoors, as much as possible, to gather more reliable data. The participants could wear a cap that shelters the frames; however, this reduces the representativeness of the task (i.e., could change movement, e.g., no heading the ball).

Through analysing the data captured during the first data collection point, it was found that the participants focused on expected environmental objects such as opponents, the ball and the goal. The data also demonstrated shared fixations, most notably between the ball and ground. These shared fixations may be acting as anchor points for more peripheral vision. Depending on the sport, task and expertise level, there is said to be three types of *gaze behaviour* [41]. As explained and illustrated by Klostermann et al. [42], a basketball free-throw allows the player to focus on one specific aspect (e.g., the basketball rim). However, in a 2 v 1 football scenario, the ball carrier’s attentional width can be increased (i.e., there is more than one cue crucial with information) and is labelled *foveal spot*. The second gaze behaviour is the *gaze anchor* as athletes locate their gaze in free space. This type of gaze behaviour allows covert attention to be used on multiple objects. However, this requires an optimal positioning of the gaze anchor [43]. The last gaze behaviour is the *visual pivot*, where gaze is located in-between relevant information sources, allowing for frequent fixation transitions. Importantly, what this recent body of work highlights within the current study is that children’s shared fixations ranged from 0.71% to 18.14% of total fixations, demonstrating the possibility of captured peripheral attention. Due to research so far focusing on static, decontextualised drills (e.g., throw and catch), this use of peripheral vision has not yet been captured or investigated in children. This finding demonstrates, for the first time, insight into what children attend to in dynamic sport environment. This finding also demonstrates that, in future research, it will be important to not only understand what children look at and for how long, but also the effective use of different types of gaze behaviours.

It also demonstrated slight differences in what children were attending to despite participating in similar coaching sessions. It is these differences that could lead to a better understanding of what children attend to, when and for how long, which can help inform teaching practice. There were only 3 fixations of the 2408 coded fixations that were not able to be coded. Two of these fixations were due to hair falling across the glasses, meaning the coder code could not see what that child was attending to. **Practical implication 2** would be to ensure that hair is secured away from the glasses.

Regarding the descriptive data of eye-gaze behaviours in this study, it should be kept in mind who the participants were. The children who participated in this study had respectable movement scores based on the TGMD-3 (FMS) and UGent assessment (motor coordination). This contextual information is not too surprising considering the participants regularly participate in football training. However, it is important to understand the participants when considering the generalisability of the implications made based on this study.

Overall, through investigating the percentage of eye gaze captured and investigating preliminary descriptive data of what 10-year-old footballers attend to, it seems this type of research method is feasible within dynamic sporting environments with a younger population. The set-up and calibration time is not too extensive; although the main barrier or constraint to this type of research, from a feasibility perspective, is the number of available portable devices and its associated cost. For larger data sets, it may be necessary to either have more than one portable device running concurrently or to have one device and run the data collection for a longer period. That being said, with one device available in this study, it was possible to capture 13 children’s eye-gaze behaviours in one 2–3-h session. This was enabled by a relatively quick turn-around of fitting, calibrating and wear time, totalling no more than 15 min per child. To ensure comparability between participants, **practical implication 3** would be to run 10 min activities, fitting the device on a child prior to the activity and only switching to the next child when the next round of that activity is about to take place. In addition, it would be beneficial to externally video the activities to compare across the eye-gaze and physical behaviour.

### 4.2. Acceptability of Using Portable Eye-Tracking Devices in Children

Overall, based on the acceptability questions supported by a framework presented by Alexandre et al. [40], most children had very little issue with wearing the eye tracker and in actuality, most issue was had with the backpack the battery pack was secured in. It was decided to place the battery pack in a backpack because of the concern around the weight of the pack when attached to children’s trousers or shorts. The concern centred around the battery being too heavy for children to have clipped onto the back of clothing, of children altering their movements due to fear of it becoming unclipped and damaged during the activities, or actually coming unclipped and damaged. Unfortunately, despite best efforts to obtain a small enough bag, due to sizing, the backpack may have been too big for some of the children’s frames, and, consequently, they experienced strap slippage. This slippage caused some distraction from the focus of the activity they were partaking in, which could lead to erroneous data capture for the eye-tracking software. Therefore, **practical implication 4** is to ensure that if the battery pack is to be fitted with a backpack, that the backpack be sized adequately for each child.

Although not many children reported the reluctance to header the ball due to the eye tracker, the fact that some did report this reluctance could also impact the decisions made by children when wearing this technology in future studies. It should be noted that the Football Association has recently banned heading the ball for children under 12 years of age for the 2022–2023 season [44]. That being said, **practical implication 5** is to clearly reassure all participating children that they do not have to alter their overall movements (e.g., body shape, speed, coordination) to avoid damage to the device either attached to the head or the torso.

Most children in this sample agreed that wearing the device was overall acceptable, and that they would be happy to wear it for longer. A couple of children did state that they would not wear it for longer and expressed minor discomfort with wearing the device. Therefore, **practical implication 6** would be to make children aware that the strap may move, the nose clip may push and the battery pack may feel a little heavy before ensuring that they are still happy to continue with wearing the device.

### 4.3. Practical Implications

Table 3 includes the six main practical implications associated with informing better procedures for designing, and for during, eye-tracking data collection.

### 4.4. Strengths, Limitations and Future Directions

Like all studies, this study has its strengths and limitations. Limitations included the small sample size; a group of thirteen 10-year-old boys is by no means a comprehensive sample, although the sample size recruited was sufficient to determine the acceptability and feasibility of eye tracking in this population. Feasibility studies such as the present study are not designed to test a hypothesis, and therefore, power calculations for such studies are not recommended. Instead, researchers recommend sample size is based upon practical considerations, including participant flow and the number of participants needed to reasonably evaluate feasibility goals [45,46]. This is an important point, as the sample was sufficient to meet the aims of the study, i.e., determining the acceptability of portable eye tracking in children. However, data collection of this nature is logistically challenging due to the cost of portable eye-tracking technology. It is therefore not feasible and cost prohibitive for multiple children to wear the eye-tracking glasses. This subsequently increases the time burden needed to collect comparable data on larger samples of participants. Alongside this, activities only involved football-related scenarios; however, football incorporates locomotor and object control related skills as well as coordination. As such, the mode of activity provided an appropriate model to determine the acceptability and feasibility of eye tracking in a dynamic sport context. We acknowledge that the data we present only reflect boys. Future work might also consider use of eye tracking in girls. The data we present in the current study were collected as part of a living lab activity with 10–11-year-old children and, as such, it was not logistically possible to extend data collection in terms of time and adding additional data collection sessions to capture additional data. An interesting next step, now that acceptability and feasibility of use has been determined would be to examine how gaze behaviour during a match context may be related to technical soccer skill, or FMS. This would require a larger sample size and different study design to achieve. A posteriori power calculation indicated that for a correlational study to detect a medium effects size of correlation, with *p* at 0.05 and power at 80%, a total sample size of 64 would be needed. However, understanding if the portable eye-tracking technology was acceptable to children and feasible to use in a dynamic sport context was essential to determine prior to larger scale with a greater analytical component being undertaken. We also assessed height, mass and sitting height to provide descriptive data on the physical characteristics of our sample. However, in the context of eye tracking, it may have been useful to also assess eye height given that there is research suggesting eye height is influential on making judgements relating to object collisions, an aspect relative to football [47]. Likewise, we did not deliberately assess scanning behaviour during the eye-tracking data period. Scanning behaviour is recognised as important in elite youth football contexts [48], but scanning per se was not particularly evident in the sample who participated in the present study. This may be because our sample consisted of non-elite grassroots players rather than elite players, and/or because the age of our sample was considerably younger (10–11-year-olds) than those where scanning has been examined (under 17–under 19-year-old elite players) [48].

The main strength of this study is in establishing the use of portable eye-tracking device with children in a representative dynamic sporting environment. This is unique in the literature and extends the work conducted in this area where activities have been primarily static or decontextualised in nature. This study is also the first to present preliminary eye-gaze data on what boys who play grassroots football attend to within football scenarios as well as the potential use of peripheral vision. This work gives the area a platform to jump off and strive towards research-informed procedures for conducting this type of research in younger populations. We importantly identify key practical implications in the use of eye-tracking technology with children which are intended to inform future research and research practice in this area.

## 5. Conclusions

This study investigated whether the use of an eye-tracking device was feasible and acceptable to use in young children during a dynamic sport task. This work found that the data gathered under certain conditions were reliable and therefore useable. When children were consulted around their experiences of wearing the portable device, this culminated in potentially important insights when using this method in future research. With better improved procedures in place, we can start to understand how best to teach children, using eye-gaze behaviour of varying ability. Ultimately, this technique could help inform teaching practice and improve motor skill learning in children, better supporting their developmental trajectories.

## Figures and Tables

**Table 1:** Descriptive statistics for football specific and movement skill assessments.

		Range	Mean (SD)	Min.	Max.
Motor coordination	UGent Soccer Specific Dribbling Test (Fastest Time (s))	-	27.71 (4.89)	20.62	34.50
FMS	Locomotor	0–24	17.23 (3.22)	13	22
	Run	0–8	6.38 (1.12)	5	8
	Jump	0–8	5.85 (1.40)	4	8
	Hop	0–8	5.00 (0.91)	4	6
	Object Control	0–22	17.31 (2.02)	14	20
	Overarm Throw	0–8	6.23 (0.83)	5	8
	Underarm Throw	0–8	6.15 (0.80)	5	7
	Catch	0–6	4.92 (0.86)	4	6
	Total FMS	0–46	34.54 (5.03)	28	42
Eye-Gaze	%	0–100	74.08 (13.27)	48	90

Note. TGMD-3 = Test of Gross Motor Development 3rd Edition, s = seconds, SD = standard deviation, Min = minimum, Max = maximum, FMS = Fundamental Movement Skills. Range = range between minimum and maximum scores for each scale.

**Table 2:** Descriptive data of what coded children attended to during 5 min of wear time.

	Number of Fixations on Environmental Objects (%)											
Participant (Coded Children)	1	2	3	4	5	6	7	8	9	10	Total Fixations	Total Saccades
1	95	136	122	94	31	51	31	1	2	0	563	172
	(16.87)	(24.16)	(21.67)	(16.70)	(5.51)	(9.06)	(5.51)	(0.18)	(0.35)			
2	31	14	90	45	178	72	0	10	0	1	441	162
	(7.03)	(3.17)	(20.41)	(10.20)	(40.36)	(16.33)		(2.27)		(0.23)		
3	60	42	120	47	132	44	1	6	2	0	454	149
	(13.21)	(9.25)	(26.43)	(10.35)	(29.07)	(9.69)	(0.22)	(1.32)	(0.44)			
4	54	50	79	54	81	139	2	2	1	0	462	136
	(11.69)	(10.82)	(17.10)	(11.69)	(17.53)	(30.09)	(0.43)	(0.43)	(0.22)			
5	96	16	114	67	148	41	0	3	0	3	488	154
	(19.67)	(3.28)	(23.36)	(13.73)	(30.33)	(8.40)		(0.61)		(0.61)		
Mean	67.2	51.6	105	61.4	114	69.4	6.8	4.4	1	0.8	481.6	154.6
SD	28	49.7	19.3	20.2	58.2	40.7	13.5	3.7	28	49.7	48.6	13.6

Note. 1 = background (fence, trees), 2 = goal, 3 = opponent/goalkeeper, 4 = coach/researcher, 5 = ground, 6 = ball, 7 = sky, 8 = boundary (line, cones), 9 = self (own hand, leg), 10 = unidentifiable.

**Table 3:** Main practical implications for informing research procedures with children.

1	Conduct eye-tracking research indoors (where possible).
2	Ensure long hair or fringes are secured so as not to obscure line of sight.
3	Run the same 10 min activity to increase comparability across children wearing the eye tracker.
4	Use a properly fitted backpack (if a backpack is to be used).
5	Assure children about the capability and hardiness of the eye tracker; they do not need to change the way they move.
6	Explain there may be some discomfort with the nose clip, head strap and battery weight and ensure that children wish to continue.

## Data Availability

Data relating to this submission are available from the primary author on reasonable request.
